# Environmental bacteriophages: viruses of microbes in aquatic ecosystems

**DOI:** 10.3389/fmicb.2014.00355

**Published:** 2014-07-24

**Authors:** Télesphore Sime-Ngando

**Affiliations:** Laboratoire Microorganismes: Génome et Environnement, UMR CNRS 6023, Clermont Université Blaise PascalAubière, France

**Keywords:** aquatic ecosystems, viruses, lysis, lysogeny, bacteria, horizontal gene transfers, food web dynamics, biogeochemical cycling

## Abstract

Since the discovery 2–3 decades ago that viruses of microbes are abundant in marine ecosystems, viral ecology has grown increasingly to reach the status of a full scientific discipline in environmental sciences. A dedicated ISVM society, the International Society for Viruses of Microorganisms, (http://www.isvm.org/) was recently launched. Increasing studies in viral ecology are sources of novel knowledge related to the biodiversity of living things, the functioning of ecosystems, and the evolution of the cellular world. This is because viruses are perhaps the most diverse, abundant, and ubiquitous biological entities in the biosphere, although local environmental conditions enrich for certain viral types through selective pressure. They exhibit various lifestyles that intimately depend on the deep-cellular mechanisms, and are ultimately replicated by members of all three domains of cellular life (Bacteria, Eukarya, Archaea), as well as by giant viruses of some eukaryotic cells. This establishes viral parasites as microbial killers but also as cell partners or metabolic manipulators in microbial ecology. The present chapter sought to review the literature on the diversity and functional roles of viruses of microbes in environmental microbiology, focusing primarily on prokaryotic viruses (i.e., phages) in aquatic ecosystems, which form the bulk of our knowledge in modern environmental viral ecology.

## INTRODUCTION

With the discovery few decades ago that viral parasites of microbes are abundant in marine ecosystems (Torrella and Morita, 1979; Bergh et al., 1989), aquatic viral ecology has increasingly grew to reach the status of full scientific discipline in environmental sciences, with the recent launch of a dedicated ISVM society, i.e., the International Society for Viruses of Microorganisms ^1^. As infectious agents of potentially all types of living cells, viruses are the most abundant biological entities in the biosphere. They are ubiquitous components of the microbial food web dynamics in a great variety of environments, including the most extreme ecosystems. Moreover, in spite of the difficulties to routinely observe and describe biological nanoparticles, combined with the absence of conserved evolution tracers such as RNA ribosomal genes, we now consider that viruses represent the greatest reservoir of non-characterized genetic diversity and resources on the earth (Suttle, 2007). They contain genes that code for essential biological functions such as photosynthesis (Lindell et al., 2005), making their hosts powerful vehicles for genetic exchanges in the environment. Because lytic viruses killed their hosts, they play fundamental roles in cycling nutrients and organic matter, structuring microbial food webs, governing microbial diversity and, to a lesser extent, by being a potential food source for protists (Sime-Ngando and Colombet, 2009). As symbionts, viruses can also form long-lived association with their specific hosts, reducing their fitness, or allowing infected hosts to remain strong competitors through mutualistic symbioses (Roossinck, 2011). In addition, the discovery and characterization of the unique group of archaeal viruses are influencing the field of prokaryotic virology, increasing our knowledge on viral diversity and changing perspectives on early stages of evolution (cf. Prangishvili et al., 2006).

Recent studies in aquatic viral ecology are source of novel knowledge related to the biodiversity of living things, the functioning of ecosystems, and the evolution of the cellular world. Viruses exhibit various life strategies that intimately depend on the deep-cellular mechanisms, and are ultimately replicated by all members of the three domains of cellular life: Bacteria, Eukarya, and Archaea. This establishes viruses as microbial killers (i.e., bad viruses) but also as cell partners and manipulators (i.e., good viruses) in the world of aquatic ecosystem (Rohwer and Thurber, 2009; Roossinck, 2011). The present chapter sought to review the literature on the diversity and functional roles of viruses in aquatic microbiology, focusing on prokaryotic viruses (i.e., bacteriophages) which form the bulk of our knowledge in aquatic viral ecology.

## DEFINITION AND LIFESTYLES

Viruses are biological entities consisting of single- or double-stranded DNA or RNA surrounded by a protein and, for some of them, a lipid coat. In aquatic systems, most viruses are tailed or untailed phages, with a capsid diameter often smaller than 250 nm, based on direct transmission electron microscopy observation. Viruses have no intrinsic metabolism and need the intracellular machinery of a living and sensitive host cell for all processes requiring energy. They have various life cycles, all starting with diffusive passive fixation on specific receptors (often transporter proteins) present at the surface of a host cell, followed by injection of the viral genome into the host cell. In the lytic cycle, the viral genome induces the synthesis of viral constituents, including the replication of the viral genetic material. A number of progeny viruses are then produced and released into the environment by the fatal rupture of the host cell.

In the chronic cycle, the progeny viruses are episodically or constantly released from the host cell by budding or extrusion, without immediate lethal events. This cycle is less well known in aquatic microbiology, but it is common in metazoan viruses such as Herpes and Hepatitis viruses or rhabdoviruses. Chronic viral infection is a dynamic and metastable equilibrium process which ends with the lysis of the host cell after serial budding of lipid membrane-coated viruses, as seen in hosts of the marine protist *Emiliania huxleyi* (Mackinder et al., 2009). Recently, chronic infection without host lysis has been reported for the first time in the marine primary producer *Ostreococcus tauri*, where the low rate of viral release through budding (1–3 viruses cell^-1^ day^-1^) allows cell recovery and the stable coexistence of viruses and their hosts (Thomas et al., 2011; Clerissi et al., 2012).

**FIGURE 1:**
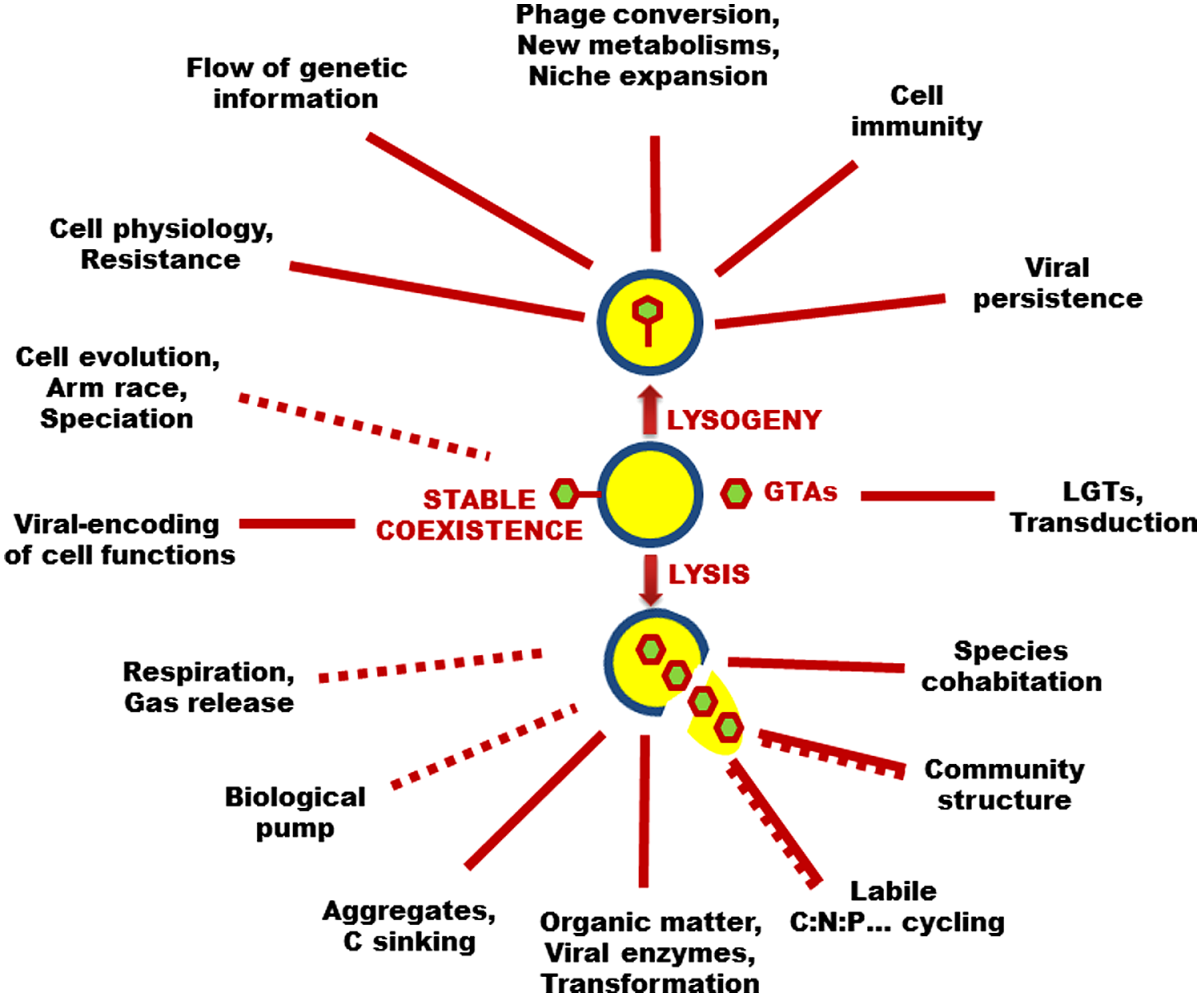
**Virus–microbe interactions range in a gradient from true non-lethal parasitism (i.e., stable coexistence) to fatal lytic infection (lysis), with intermediate mutualistic lifestyles (lysogeny and pseudolysogeny).** Because of the existence of such a large panel of lifestyles and in conjunction with the fact that all types of cells are sensitive to unique viruses, these biological entities are considered the most diverse, abundant, and ubiquitous biological entities in the biosphere where they have tremendous effects on the diversity of living things, the functioning of microbial ecosystems, and the evolution of the cellular world. Some of these direct (solid lines) and indirect (dashed lines) effects on aquatic microbial processes are highlighted in this figure. Please refer to the main text for abbreviations.

In the lysogenic cycle, the viral genome integrates the genome of the host cell and reproduces as a provirus (or prophage) until an environmental stress to the immune host cell sets off a switch to a lytic cycle. Both the provirus and the host cell benefit from lysogeny. Lysogeny provides a means of persistence for viruses when the abundance of the host cells is very low. Prophages may affect the metabolic properties of host cells which can acquire immunity to superinfections and new phenotypic characteristics such as antibiotic resistance, antigenic changes, and virulence factors, resulting in niche expansion for viral hosts (**Figure 1**). A variant to the lysogenic cycle is the so-called carrier state or pseudolysogenic cycle, where the viral genome is not integrated with the host genome but rather remains in an “inactive state” within the host cell. There is no replication of the viral genome which is segregated unequally into progeny cells, most likely for a few generations. Pseudolysogenic viruses probably occur in very poor nutrient conditions where host cells are undergoing starvation and cannot offer the energy necessary for viral gene expression.

## TAXONOMY

There are three domains of life – Archaea, Bacteria, and Eukarya – that consist only of cellular organisms (Woese et al., 1990). Because viruses lack the ribosomal RNA nucleotide sequence upon which these cellular domains of life are based, they cannot be integrated into the cellular tree of life (Breitbart et al., 2002; Rohwer and Edwards, 2002), although susceptibility to virus infection is a common feature of all members of the three domains of life. In the absence of universal evolution markers for the entire viral world, viruses have been grouped by many different methods, according to various criteria: the nature of the host, the characteristics of the free virions (phenotype, genotype, resistance to organic solvents for viruses with lipid coat, etc.), or even the name of the related illness, the laboratory or the researcher working on the targeted viruses. The 9th report by the International Committee on Taxonomy of viruses (ICTV) ^2^ includes 6 orders, 87 families, 19 subfamilies, 349 genera, and 2284 virus and viroid species, defined as a group of viruses that constitutes a replicative lineage and occupies a particular ecological niche (Regenmortel, 1992). Hurst (2011) introduced the idea that the taxonomy of viruses and their relatives could be extended to the domain level, and suggests the creation of an additional biological domain that would represent the acellular infectious agents that possess nucleic acid genomes or the genomic acellular agents. The proposed domain title is Akamara, whose derivation from the Greek (a + kamara) would translate as “without chamber” or “without void.” The domain is divided into two kingdoms. The kingdom Eurivia includes true viruses and those satellite viruses whose genomes code for their own capsid proteins, and is separated in two Phyla (RNA and DNA viruses). The second kingdom (Viroidia) forms one phylum that includes viroids along with other groups of related agents whose genomes likewise do not code for their structural “shell” proteins.

A new way to classify phages has also been proposed based on the complete sequences of 105 viral genomes. This so-called phage proteomic tree places phages relative to their neighbors and all other phages included in the analysis, which is a method that can be used to predict aspects of phage biology and evolutionary relationships, and to highlight genetic markers for diversity studies (Rohwer and Edwards, 2002). The approach is useful for those phages whose complete genomes have been deciphered (i.e., a minority of environmental viruses); primarily those belonging to a common pool of genes (e.g., following genetic recombination), or which have evolved from a common ancestor. It is well known that the genomes of some phages, if not most, are mosaics of genes from various sources, including other phages and their hosts (Hendrix et al., 1999).

## PHENOTYPIC TRAITS

The first descriptions of the global diversity of viruses are from the general forms of virus-like particles observed via transmission electron microscopy. In aquatic samples, viral phenotypes are limited, mainly including tailed or untailed particles with capsid heads, characteristics of bacteriophages. Tailed phages belong to the order Caudovirales, all of which are double-stranded DNA viruses that generally represent 10–40% of the total abundance of viruses in aquatic systems (see comparative tables in Wommack and Colwell, 2000; Sime-Ngando and Colombet, 2009). Within Caudovirales, three families emerge as quantitatively dominant: Siphoviridae with long, non-contractile tails (e.g., Phage lambda), Podoviridae with a short, non-contractile tail (e.g., Phage T7), and Myoviridae with contractile tails of variable length (e.g., Phage T4). In most studies, non-tailed capsids dominate viral abundances. This may be an artifact due to the effects of mechanic shocks resulting from handling, primarily ultracentrifugation (Colombet et al., 2007), because 96% of the 5500 specimens of described bacteriophages are tailed particles (Ackermann, 2007). However, a recent global morphological analysis of marine viruses suggested that non-tailed viruses, which comprised 50–90% of the viral particles observed, might represent the most ecologically important component in natural viral communities (Brum et al., 2013).

Phenotypic traits and viral morphs in aquatic viruses are cryptic of the selective pressures faced by these communities, and provide insight into host range, viral replication and function (Suttle, 2005). For instance, myoviruses are mostly lytic with a large spectrum of sensitive hosts, which is a competitive advantage that can be assimilated to r-strategist species thriving with high proliferation rates in fluctuating environments. In contrast, podoviruses are more highly specific to their hosts, with siphoviruses being intermediate between myo- and podoviruses. In addition, several siphoviruses can encode their genome into their hosts for several generations (i.e., lysogeny), which can be rather assimilated to K-strategist species, characteristics of stable environments. Combined with the capacity of viruses to potentially face almost all types of environments and the related interfaces (Hurst, 2011), the ability of viruses to develop along the r-K-selection continuum, i.e., from typical r (e.g., prokaryotes) to K (e.g., vertebrates) strategists (Suttle, 2007), may help to explain their ubiquity, hence the notion of the virosphere (i.e., viral biosphere).

## GENOMIC DIVERSITY

ICTV-reported viral species are mostly known from their isolated hosts in laboratory cultures which, in the case of environmental samples, may not exceed 1% of the total prokaryotes (Hugenholtz et al., 1998). This implies that the diversity of environmental viruses is huge, although the bulk of the estimated 10^31^ viruses in the biosphere is unknown (Rohwer and Edwards, 2002). In addition to whole genome sequencing of specific phages (Allen et al., 2011) or genomes assembled from metagenomics datasets (Rosario et al., 2009), molecular approaches applied to uncultured complex communities are thus critical and offer windows to the largest uncharacterized reservoir of diversity on the earth (Hambly and Suttle, 2005). Polymerase chain reaction (PCR)-based methods are restricted to chosen viral groups as no gene is universally conserved among viruses, while part of the existing diversity of these viral groups is missed because PCR primers are based on previously identified sequences described in public databases. Viral metagenomics gives access to an exhaustive view of uncultured viral diversity (Breitbart et al., 2002), and has so far revealed an important unknown diversity and an unexpected richness of viral communities (Edwards and Rohwer, 2005).

Despite the ecological importance of viruses, a fundamental hindrance to their integration into microbial ecology studies is the lack of suitable reference bacteriophage genomes in reference databases. Currently, only eight bacterial phyla (Proteobacteria, Firmicutes, Bacteriodetes, Actinobacteria, Cyanobacteria, Chlamydiae, Tenericutes, and Deinococcus-Thermus) of 29 phyla with cultured isolates have sequenced phage representatives, contributing only 0.001% of genome sequence databases (Bibby, 2014). From these few phage genomes, comparative genomics have revealed an impressive level of genomic diversity and novelties as well as hypotheses on potential adaptation of phage genome to aquatic environments. For example, comparison of 26 T4-like genomes of myoviruses infecting diverse marine cyanobacteria (*Prochlorococcus* or *Synechococcus*) has revealed highly syntenic hierarchical cores of genes, with DNA replication genes observed in all genomes, followed by previously described, virion-structural and various hypothetical genes. Beyond previously described cyanophage-encoded photosynthetic and phosphate stress genes, genes involved in various putative functions (e.g., phytanoyl-CoA dioxygenase, 2-oxoglutarate) were indeed described, as well as non-core genes that may drive complex niche diversification (Sullivan et al., 2010). The unveiling of the first genome of a deep-photic marine cyanobacterial siphovirus highlighted the prevalence of lysogenic lifestyle and significant divergence and size differences with previously sequenced siphoviruses, and the absence of photosynthetic genes which have consistently been found in other marine cyanophages (Sullivan et al., 2009). Similarly, the genomic and functional analysis of a novel marine siphovirus of marine *Vibrio* revealed a larger genome (80,598 bp) compared to that of most known siphoviruses, with a novel shell symmetry that confers a remarkable stability to a variety of physical, chemical, and environmental factors (Baudoux et al., 2012). Whole genome sequencing/reconstruction of phages that are currently unrepresented in the database will thus likely provides deep insights into and have a significant impact on our view of viral diversity, ecology, and evolution, while providing molecular tools for the study of groups of viruses.

Whole genome comparisons indeed have also shown that there are conserved genes shared among all members within certain viral taxonomic groups. These conserved genes can be targeted using PCR amplification and sequencing for diversity studies of groups of cultured and environmental viruses. Examples of such genes are structural proteins such as gp20, which codes for the capsid formation in T4 phage-like viruses, DNA polymerases for T7-like podophages, or the RNA-dependent RNA polymerase fragment, which has been used to identify novel groups of marine picornaviruses (Culley et al., 2003). All of the conserved gene studies suggest that environmental viral diversity is high and essentially uncharacterized (Breitbart and Rohwer, 2005).

For the whole environmental communities, molecular fingerprinting approaches that separate PCR-generated DNA products, such as denaturing gradient gel electrophoresis (DGGE, Short and Suttle, 2002) and pulse-field gel electrophoresis (PFGE, Wommack et al., 1999), have been widely used but with limited results, restricted to double-stranded DNA viruses. With this approach, the genome size of aquatic viruses fluctuates from 10 to about 900 kb, with mean ranges of 10–630 kb and 10–660 kb in marine and freshwater systems, respectively. The frequencies of the distribution of genome size classes are multimodal, with peaks in the interval < 70 kb and a mean at 50 kb.

An introduced fingerprinting approach adapted to viruses, randomly amplified polymorphic DNA-PCR (RAPD-PCR), allows sampling of viruses at the genetic level without requiring viral isolation or previous sequence knowledge (Winget and Wommack, 2008). RAPD-PCR is accurate in assessing DNA viral richness in water samples by using single 10-mer oligonucleotide primers to produce amplicons (PCR-generated DNA fragments) and banding patterns, with each likely representing a single amplicon that originates from viral template DNA. Such an approach has been demonstrated to match observations from other community profiling techniques, revealing more temporal than spatial variability in viroplankton assemblages. Hybridization probes and sequence information can also be easily generated from single RAPD-PCR products or whole reactions, providing a tool for routine use in high-resolution viral diversity studies by providing assemblage comparisons through fingerprinting, probing, or sequence information.

Metagenomics has revolutionized microbiology by paving the way for a culture-independent assessment and exploitation of microbial and viral communities present in complex environments (Simon and Daniel, 2011). Metagenomic viral analyses or virome studies suggest that environmental viral diversity is high and essentially uncharacterized (Angly et al., 2006; Roux et al., 2012). Metagenomic analyses of 184 viral assemblages collected over a decade and representing 68 sites in four major oceanic regions showed that most of the viral DNA and protein sequences were not similar to those in the current databases (Angly et al., 2006). Global diversity was very high, presumably several hundred thousand species, and regional richness varied on a North–South latitudinal gradient. However, most viral species were found to be widespread, supporting the idea that marine viruses are widely dispersed and that local environmental conditions enrich for certain viral types through selective pressure. A study on comparative viral metagenomics highlighted that freshwater, marine, and hypersaline environments were separated from each other despite the vast geographical distances between sample locations within each of these biomes, suggesting a genetic similarity between viral communities of related environments (Roux et al., 2012).

Interrogation of microbial metagenomic sequence data collected as part of the Sorcerer II Global Ocean Expedition (GOS) also revealed a high abundance of viral sequences, representing approximately 3% of the total predicted proteins in the 0.1–0.8 μm size fraction of the plankton (Williamson et al., 2008). Viral sequences revealed hundreds to thousands of viral genes, encoding various metabolic and cellular but mostly structural functions. Quantitative analyses of viral genes of host origin confirmed the viral nature of these sequences and suggested that significant portions of aquatic viral communities behave as reservoirs of such genetic material. Distributional and phylogenetic analyses of these host-derived viral sequences also suggested that viral acquisition of environmentally relevant genes of host origin is a more abundant and widespread phenomenon than previously appreciated. The predominant viral sequences identified within microbial fractions originated from tailed bacteriophages and exhibited varying global distributions according to viral family. The recruitment of GOS viral sequence fragments against 27 complete aquatic viral genomes revealed that only one reference bacteriophage genome was highly abundant and was closely related, but not identical, to the cyanobacterial myovirus P-SSM4 of *Prochlorococcus* hosts, suggesting that this virus may influence the abundance, distribution, and diversity of one of the most dominant components of small phytoplankton in oligotrophic oceans (Williamson et al., 2008).

Overall, metagenomic analysis of viruses increasingly suggests novel patterns of evolution, changes the existing ideas on the composition of the virus world, and reveals novel groups of viruses and virus-like agents (Kristensen et al., 2010). The gene composition of marine DNA viromes is dramatically different from that of known bacteriophages. The virome is dominated by unknown genes, many of which might be contained within virus-like entities such as gene transfer agents (GTA), which are host DNA carrier particles (Lang et al., 2012). Analysis of marine metagenomes thought to consist mostly of bacterial genes revealed a variety of sequences homologous to conserved genes of eukaryotic nucleocytoplasmic large DNA viruses, resulting in the discovery of diverse members of previously undersampled groups and suggesting the existence of new classes of virus-like agents.

Unexpectedly, metagenomics of marine RNA viruses showed that representatives of one superfamily of eukaryotic viruses, the picorna-like viruses, dominate the RNA virome (Kristensen et al., 2010). Marine RNA viruses are almost exclusively composed of those that infect eukaryotes (Lang et al., 2009), primarily protists (Culley et al., 2007). This was confirmed in a recent quantitative study where the comparison of the total mass of RNA and DNA in viral fraction suggests that the abundance of RNA viruses equaled or exceeded that of DNA viruses in coastal seawater (Steward et al., 2013). Similar findings were also reported in freshwater systems (Roux et al., 2012). Because picorna-like viruses have small genomes, they are at or below the detection limit of common fluorescence-based counting methods, implying that protists contribute more to marine viral dynamics than one might expect based on their relatively low abundance. Similarly, a recent metagenomic study from temperate and subtropical seawater has highlighted 129 genetically novel and distinct viruses based on complete genome assemblages, all of which were single-stranded DNA viruses mostly known as economically important pathogens of plants and animals (Labonté and Suttle, 2013). The discovery of RNA and ssDNA viruses is a significant departure from the prevailing view of aquatic viruses which are assumed to mostly contain double-stranded DNA and infect bacteria. It is thus likely that we are missing a significant fraction of viruses in aquatic ecosystems, which probably is one of the many reasons that may help explain the apparent discrepancy between genome-derived and metagenome-derived diversity of viruses. These reasons are highlighted in Ignacio-Espinoza et al. (2013).

## ABUNDANCE, DISTRIBUTION, AND BIOGEOGRAPHY

Viruses were first suspected as abundant particles in the sea in the late 1970s (Torrella and Morita, 1979), which was confirmed one decade later with the discovery that 1 ml of sea water contains millions of viruses (Bergh et al., 1989). First estimates were variable and inaccurate because they were based on manipulated (i.e., ultracentrifuged) samples observed at high magnification using transmission electron microscopy. More accurate and reproducible estimates were provided later using direct epifluorescent microscopy or flow cytometry, yielding viral abundances that exceed those of Bacteria and Archaea by an overall average of about 15-fold (Bettarel et al., 2000). It is important to use fluorochrome dyes (e.g., SYBR Gold) that bind to ssDNA and ARN viruses which have been proved, based on metagenomics, to be much more abundant in natural aquatic systems than expected (Lang et al., 2009; Holmfeldt et al., 2012). Furthermore, in this context of methodological difficulties for accurate estimates of the numerical abundance of natural viruses, it is important to stress the recent discovery that DNA associated with membrane-derived vesicles, gene transfer agents, or cell debris can produce fluorescent dots that can be confused with viruses (Forterre et al., 2013). This targets a critical problem that needs to be bypassed in the future because many bacterial species, including the dominant marine cyanobacterium *Prochlorococcus* spp., release extracellular vesicles which have roles in various processes (e.g., quorum sensing, virulence, horizontal gene transfers, etc.), and were recently demonstrated to have a significant effect on carbon cycling in marine ecosystems (Biller et al., 2014).

Viral abundance generally increases with the increasing productivity of aquatic ecosystems and, as a consequence, decreases from freshwater to marine ecosystems, from costal to oceanic zones, and from the surface to the bottom of the euphotic layer (Sime-Ngando and Colombet, 2009). The abundance of viruses in individual aquatic systems appears to be independent of salinity but related to the biomass of primary and secondary producers, as well as to seasonal effects (Wilhelm and Matteson, 2008). In the dark ocean (i.e., meso- and bathypelagic zones), where about 75% of prokaryotic biomass and ca. 50% of prokaryotic carbon production in the world ocean occur (Aristegui et al., 2009), high abundance of viruses was observed (Parada et al., 2007). Similarly, two deep marine sediment studies from Ocean Drilling Project samplings in Saanich Inlet, Canada (Bird et al., 2001), and on the Eastern margin of the Porcubine Seabight (Middelboe et al., 2011) have reported abundant viruses and prokaryotes in >100 m sediment cores aged from 0 to 14,000 years and from 0.5 to 2 million years, respectively. On a volumetric basis, viral abundances in sediments exceed 10–1000 times that in the water column, representing active and mostly endemic components of benthic environments (Danovaro et al., 2008), although visibly infected cells are often scarce (Filippini et al., 2006). Because the relative abundances of Archaea increase in the dark deep ocean and freshwater lakes, viruses of Archaea are also expected to be abundant there, as recently suggested by highly complex, diverse morphologies observed in a deep-dark permanently anoxic freshwater lake, some of which being putatively new for science (Borrel et al., 2012). Thus, it is likely that the ecology of the deepest and benthic waters where eukaryotes are constrained by poor oxygen conditions is essentially driven by the dark viral loop (dissolved organic matter–prokaryotes–viruses) processes (Colombet and Sime-Ngando, 2012).

According to time, viral abundances fluctuate on diverse scales, from minutes to years, often in association with prokaryotes, which offer the major reservoir for hosts (Wommack and Colwell, 2000). Surprisingly, there is only one to two orders of magnitude variation in virus abundance among systems (c. 100-fold; 10^9^–10^11^ virus particles l^-1^) in spite of more than three orders of magnitude variation in the planktonic biomass, as one ranges from either coastal to offshore or from surface to deep-water environments (Wilhelm and Matteson, 2008). This may help to explain why the virus-to-prokaryote ratios (VBRs) fluctuate substantially, with an overall increase from 3 to 10 in oligotrophic marine systems to 6–30 in productive freshwaters where the burst size (i.e., the number of viruses release per lyzed host cell) and the contact and infection rates are generally higher (Sime-Ngando and Colombet, 2009). The higher VBRs in productive lakes may also reflect the increasing relative abundance of non-bacteriophage viruses along the trophic gradient of aquatic systems (Bettarel et al., 2003). Virus abundances in freshwaters appear to vary more strongly on seasonal scales than in marine environments, especially in lakes that undergo pronounced seasonal cycles, although the linkages between seasonal cycles and virus abundance remain unresolved in the absence of long-term studies (Wilhelm and Matteson, 2008). Evidence that viral abundance across oceans and lakes is driven by different factors was provided based on case studies, including bacterial and cyanobacterial abundances, and chlorophyll-*a* concentration as significant variables in lakes, bacterial and cyanobacterial abundances for coastal Pacific Ocean, and bacterial abundance and chlorophyll-*a* concentration for coastal Arctic Ocean (Clasen et al., 2008). However, this mainly concerns free-floating viruses in the water column. Methodological progress based on confocal laser scanning microscopy in combination with lectin and nucleic acid staining has demonstrated that viruses trapped in organic aggregates are much more abundant than in the water column, ranging from 10^8^ to 10^14^ viruses l^-1^. Organic aggregates and inorganic particles appear to play a role of viral scavengers or reservoirs rather than viral factories, and can enhance the growth rates of free-living prokaryotic community. The problems and knowledge gaps in virus–particle interactions, and the related research avenues and implications for water-column ecological processes (e.g., microbial diversity, food web structure, biological pump, biogeochemical cycles, etc.), are provided in an excellent review by Weinbauer et al. (2009).

On a global scale, the forces that shape the biogeography of viruses have received very little attention. It is of interest to search for general patterns of microbial and viral biogeography because general ecological theories, actually known solely from “macroscopic” or visible species (e.g., the positive relationship between diversity and area sampled, or the negative one between local abundance and body size), will offer predictive tools in the context of global change. Microorganisms and their viruses have long been considered as ubiquitous, without a biogeography of any sort. This is because their dispersal is thought to be unlimited due to small size, large absolute abundances and the formation of resistant or dormant stages. The ubiquity tenet for microorganisms is the so-called Baas Becking statement “*everything is everywhere, but the environment selects*” (Fontaneto, 2011). The assumption that viruses are ubiquitous across habitats is currently being evaluated and some phages could be globally distributed, while others could be unique and perhaps endemic to specific habitats (Roux et al., 2012), primarily to extreme environments such as deserts (Prigent et al., 2005; Prestel et al., 2008) or deep-dark permanently anoxic volcanic lake sediments (Borrel et al., 2012). It was also extrapolated from metagenomic data that viral diversity could be high on a local scale but relatively limited globally, and that viruses promote horizontal gene transfers by moving between environments (Breitbart and Rohwer, 2005). Further work is required to fully resolve and confirm the drivers of viral large-scale distribution, in conjunction with the improvement of taxonomy, methods, and sampling effort for both viruses and their hosts.

## LYTIC ACTIVITY, MICROBIAL MORTALITY, AND BIOGEOCHEMICAL IMPLICATIONS

The diversity and the abundance of total viruses are not always correlated to the lytic activity. Most free-occurring viruses are considered infectious (Suttle, 2005). It is now well accepted that lytic viruses represent one of the main causes of microbial mortality in aquatic systems (**Figure 1**). Based on the direct observation of infected cells, viral-mediated mortality averages 10–50% of the daily production of heterotrophic prokaryotes and approximately equals the bacterivory from grazers in both fresh and marine waters (Fuhrman and Noble, 1995; Pradeep Ram et al., 2005). These values fluctuate largely (from zero to near 100%) depending mostly on the host availability (density and activity), although physicochemical factors such as solar radiations (Wilhelm et al., 1998), temperature (Pradeep Ram et al., 2005) or anoxia (Colombet et al., 2006) can impact the lytic activity of viruses. The populations of lytic viruses ultimately depend on the availability of specific hosts, and could thus respond to the growth rate of the most active hosts. This pattern has the strong feedback effect of preventing species dominance and enhanced species cohabitation within microbial communities, i.e., the so-called phage kills the winner hypothesis (Thingstad and Lignell, 1997). Viral lytic activity was also demonstrated as uneven and heterogeneous for different prokaryotic phenotypes and/or genotypes, a situation which can shape the host diversity and community structure, and thus exert a strong influence on the processes occurring in the plankton food web dynamics (Pradeep Ram et al., 2010).

Because viruses kill microbial hosts which dominate the biological biomass in pelagic systems, including bacteria, archaea, cyanobacteria, protists, and fungi as major partners, they have an overwhelming effect, both directly and indirectly, on the cycling of the major conservative elements (C, N, P, etc.) upon which the food web dynamic is based (Fuhrman, 1999; Wilhelm and Suttle, 1999). It was estimated that the absolute abundance of oceanic viruses results in about 10^29^ infection events day^-1^, causing the release of 10^8^–10^9^ tons of carbon day^-1^ from the living biological pool (Suttle, 2007). By exploding microbial cells, lytic viruses are strong catalyzers of the transformation of living organisms to detrital and dissolved phases available to non-infected microbes. This biogeochemical reaction increases the retention time of organic matter and its respiration in the water column and weakens the trophic efficiency of the food web, but also provides nutrients (e.g., directly or indirectly from mineralization and photodegradation of dissolved organic matter, DOM) to primary producers (**Figure 1**; Weinbauer, 2004). For example, it has been shown that the iron contains in the viral lysis products could fulfill the metabolic requirements of marine phytoplankton (Poorvin et al., 2004). Primary production in the size fraction 2–200 nm can be depleted by about half due to an increase in viral abundance as low as 20%. A modeling exercise suggested that viral lysis of 50% of bacterial production could increase microbial respiration by 27%, while decreasing the grazing efforts from protists and metazoan zooplankton by 37 and 7%, respectively. When adding 7% of viral-mediated loss of phytoplankton and 3% grazing of viruses by phagotrophic flagellates, bacterial respiration increases to 33% (Fuhrman, 1999).

The effects of lytic viruses thus directly affect DOM concentration but also its composition. For example, Lønborg et al. (2013) recently demonstrated that viral lysate from an axenic culture of *Micromonas pusilla* significantly change the DOM composition by increasing the amounts of transparent exopolymer particles (TEP), aromatic amino acids, and humic DOM, with an elevated protein : humus ratio that suggested a higher contribution of labile components in viral-produced DOM than in algal exudates. At the natural community level, these results suggested that viral lysis could decrease the organic matter sedimentation and promotes respiration and nutrient retention whereas, in contrast, the enhanced TEP production could stimulate particle aggregation and export out of the water column. Based on metabolomics approach, Ankrah et al. (2014) demonstrated that phage infection of *Sulfitobacter* sp. redirects 75% of nutrients into virions, and 71% of 82 intracellular metabolites were significantly elevated in infected hosts, which also exhibited an elevated metabolic activity compared to non-infected populations. In contrast, more than 70% of 56 compounds in viral lysate decreased in concentration relative to uninfected controls, suggesting that small, labile nutrients from viral lysis are utilized quickly by non-infected cells. Ankrah et al. (2014) conclude that virus-infected cells are physiologically different from their uninfected counterparts, a situation which can alter the ecosystem biogeochemistry. One of the intrinsic mechanisms for that is the viral control of bacterial growth efficiency over a broad range of values (from 0.1 to 70%) and the related patterns in carbon and nutrient fluxes mediated by bacteria in pelagic environments (Bonilla-Findji et al., 2008; Motegi et al., 2009; Pradeep Ram et al., 2013).

There are many indirect ways that viruses can affect the biogeochemical cycling. Lytic viruses may shape the global climate by inducing the release of dimethyl sulfide (C_2_H_6_S), a gas known to influence cloud nucleation (**Figure 1**), which is massively produced by major bloom-forming species such as *M. pusilla*, *E. huxleyi*, and *Phaeocystis pouchetii* (Evans et al., 2007). Viral lysis of microorganisms in sinking aggregates could also decrease the sinking rate by alleviating the aggregates via release of trapped dissolved and colloidal materials. Alternatively, virus-infected cells could sink faster compared to non-infected cells (Lawrence and Suttle, 2004), contributing to the export of microbial cells downwards (**Figure 1**). In addition, viral lysis products contain polymers that can increase gel formation and affect the biological, physicochemical and optical properties of the sea water (Uitz et al., 2010), for example by generating aggregates and enhancing the departure of organic material from the euphotic zone (Lønborg et al., 2013). This can influence the amount of carbon exported to the deep ocean by the so-called biological pump, but also the Redfield stoichiometry of the water column, because the export of nutrients other than carbon needs to be balanced by new inputs (Mari et al., 2005). Hence, highly labile N- and P-nutrients contained in nucleic acids and amino acids, for example, will be used rapidly and retained in the euphotic layer, while the sinking particles will be abnormally rich in carbon, thereby increasing the efficiency of the oceanic biological pump activity (**Figure 1**; Suttle, 2007).

## LYSOGENY: A SURVIVAL STRATEGY FOR VIRUSES

One of the key explanations for the omnipresence of viruses in natural ecosystems is undoubtedly through the existence of several lifestyles, of which two major pathways, namely lysis and lysogeny, are prevalent in aquatic systems (Pradeep Ram and Sime-Ngando, 2010). Lytic infections are by far the best studied of the virus–host interactions. Lysogenic activity has been less studied in aquatic environments where the temperate phage can alternatively integrate into the host genome as prophage. Prophages can be stable in their host for long periods of time, from months to years, with low probability of bacteriophages being released by spontaneous lysis (Paul, 2008). Examinations of natural prokaryotic communities inducible with a mutagenic agent (e.g., mitomycin C) have suggested that the fraction of lysogenic bacteria is typically <50% (range 0–100%) of the total abundance in marine environments. In freshwaters, these values fluctuate from 0 to 16% in temperate and tropical lakes, and from 0 to 73% in Antarctic lakes (Sime-Ngando and Colombet, 2009).

Lysogenic conversion has been described as a means of survival for viral populations that are threatened by poor host cell abundance and therefore cannot sustain population numbers through lytic infection alone (Stewart and Levin, 1984; Palesse et al., 2014). This situation occurs when the prokaryote abundance drops under a minimal threshold level, typically under about 10^5^ cells ml^-1^ (Pradeep Ram et al., 2005). Microcosm experiments have recently demonstrated that nutrient addition in freshwater samples stimulates lytic viruses via enhanced growth rate of prokaryotes and, when limiting, rather promote lysogenic conversion (Pradeep Ram and Sime-Ngando, 2010). This finding was considered an explanation why lytic and lysogenic activities are often antagonistically correlated, supporting the idea that lysogeny may represent a maintenance strategy for viruses in harsh nutrient/host conditions which appeared as major instigators of the trade-off between the two viral lifestyles (Colombet et al., 2006). Both viral life cycles are thus apparently regulated by distinct factors, including environmental parameters (primarily resources for hosts) and host physiology for lytic cycle but mainly host physiology for lysogenic cycle (Maurice et al., 2013; Palesse et al., 2014). It was also shown that the latter cycle is prevalent within the phylogenetic groups that dominate the whole bacterial community composition at a given time (Maurice et al., 2011).

## VIRUSES AND MICROBIAL DIVERSITY

Viruses can impact microbial diversity and force diversification mechanisms toward host-cell evolution in two major ways (**Figure 1**). The first major way includes the direct effects of the intrinsic activities of viruses: (i) keep in check competitive dominants (i.e., lytic viruses), (ii) affect the metabolic properties of host cells which can acquire immunity to superinfections and new phenotypic and genotypic traits such as production of toxins (i.e., temperate phage conversion), and (iii) transfer both viral and host genes between species (transduction, GTA), thereby influencing speciation. The second major way comprises the indirect effects of viral activities such as (iv) the structuring effects of lysis products on species composition and richness, (v) the sustenance of the amount of information encoded in genomes that may favor horizontal gene transfer mechanisms, and (vi) the effects of physiological mechanisms involved in the resistance of host against viruses, through the host-pathogen arms race (**Figure 1**). Together with (i) the high abundance and broad geographical distribution of viruses and viral sequences within microbial fractions, and (ii) the prevalence of genes among typical viral sequences that encode microbial physiological functions, the above-mentioned effects establish environmental viruses as strong vectors that generate genetic variability of aquatic microorganisms and drive both ecological functions and evolutionary changes (Weinbauer and Rassoulzadegan, 2004).

### VIRAL LYSIS PRODUCTS SHAPE EVOLUTIONARY TRANSITION, GLOBAL CARBON CYCLING AND THE DIVERSIFICATION OF MICROBIAL COMMUNITIES

Some viral groups such as the Caudovirales, the tailed double-stranded DNA phages, are probably older than the separation of life into the three now recognized domains of life (Ackermann, 1999; Hendrix, 1999). This suggests that, before the occurrence of eukaryotic grazers such as flagellates and ciliates, viruses were probably the main predators of cells in the prokaryotic world, and played a major role in the sophisticated forces (dispersal, competition, adaptive radiation, etc.) that shape biogeography and evolution. In contemporaneous marine systems, it was estimated that between 6% and 26% of the photosynthetically fixed carbon is channeled or “shunted” to the DOM pool by viral lysis of cells at all trophic levels (Wilhelm and Suttle, 1999). The carbon stored in the oceanic DOM pool equals that in atmospheric CO_2_ (Hedges, 1992), suggesting that viral infection of marine prokaryotes and phytoplankton has an influence not only on global carbon cycling and climate but also on the microbial composition and community structure. A host density-dependent model, i.e., “the phage kills the winner” model (Thingstad and Lignell, 1997; Thingstad, 2000), was proposed based on the assumption that lytic viruses are highly specific to their host cells, at least at the species level. In this model, by eliminating the most competitive strains for resource acquisition, lytic viruses prevent dominance and increase niche availability for species-coexistence (**Figure 1**). This was recently tested by Motegi et al. (2013) who provide experimental data showing that viruses prevent the prevalence of taxa that were competitively superior in phosphate-replete conditions in NW Mediterranean surface waters. In addition, these authors obtained a statistically robust dome-shaped response of bacterial diversity to viral (VP) to bacterial (BP) production ratio, with significantly high bacterial diversity at intermediate VP:BP, corroborating the prediction from the general model that species diversity is maximized when productivity and disturbance are balanced.

### LYSOGENS AND THE EFFECTS OF THEIR MUTUALISTIC VIRUSES

Virus-host interactions range in a gradient from true non-lethal parasitism (i.e., chronic infection) to fatal lytic infection, with intermediate mutualistic lifestyles (lysogeny, pseudolysogeny) where viral genomes stay within the host and confer new metabolic traits which can increase the fitness of the immune host but also the survival of the phage (**Figure 1**). A spectacular case for such a phage conversion is the finding that cholera infection is due to a lysogenic strain of the *Vibrio cholerae* bacterium. Since the toxin is encoded in the genome of the prophage and is not part of the host genome, non-lysogenic cells do not cause cholera (Weinbauer, 2004). In addition, it was recently shown that the type VI secretion systems in *V. cholerae* are virulence-associated proteins that are evolutionarily related to components of bacteriophage tails (Basler et al., 2012). Prophage induction events can change the bacterial community structure by increasing the diversity and richness of natural bacterial populations (Hewson and Fuhrman, 2007). For example, phage conversion can increase the fitness of cells (Edlin et al., 1975; Lin et al., 1977), which could influence community composition by allowing for the survival or dominance of such converted cells. Host immunization against infection by homologous phages and phage conversion are ways in which phages can influence microbial diversity in natural environments. More generally, it is likely that all living cells could contain active prophages in their genome. On average, 2.6 prophages have been detected per free-living bacterial species (Lawrence et al., 2002), and a number of bacterial genomes contain between 3 and 10% of DNA prophages (Brüssow and Hendrix, 2002). It was recently demonstrated that genes captured from ancestral retroviruses have been pivotal in the evolutionary acquisition of the key process through which most of the maternofoetal exchanges take place in placenta development in mammalian species (Dupressoir et al., 2009). This highlights the potential of phages as key players in the evolution and maintenance of living things.

### LATERAL GENE TRANSFERS (LGTs) BY TRANSDUCTION: VIROMES ARE HUGE RESERVOIRS OF VIRALLY ENCODED HOST GENES THAT ARE CANDIDATES FOR LGTs

One of the most surprising findings of whole-genome sequencing is the enormous extent of lateral gene transfers. LGTs refer to the gene material exchanges between organisms that happen independently of reproduction (i.e., vertical gene transfers). General mechanisms include transformation (gene transfer by uptake of free genetic materials), conjugation (direct gene transfer from cell-to-cell contact), the activity of GTAs, and transduction, where viruses are the main vectors that move nucleic materials from one cell to another (**Figure 1**). The transduction frequency in natural waters ranges from 10^-8^ to 10^-5^ per virus, and they might be up to 100 transductants l^-1^ day^-1^ (Jiang and Paul, 1998). An extrapolation exercise suggests that as many as 10^24^ genes are moved by transduction from viruses to hosts each year in the world’s ocean (Rohwer and Thurber, 2009). This is considered an underestimate because of the action of the host DNA carrier GTAs (primarily in the α-Proteobacteria order Rhodobacterales) which are injected into recipient cells, providing a more efficient form of transduction (Lang et al., 2012). Although transduction is a random process, viruses can genetically alter microbial populations through lysogeny and transduction, and affect the flow of genetic information in aquatic ecosystems.

Large-scale metagenomics has shown that viruses contain diverse genes of interest, including virulence genes such as the cholera toxin genes, respiration, nucleic-acid, carbohydrate and protein metabolism genes, as well as genes involved in vitamin and co-factor synthesis, in stress response, and in motility and chemotaxis, which are more common in viromes (metagenomes of viruses) than in their corresponding microbiomes (metagenomes of microbes; Rohwer and Thurber, 2009). Microbes that take up these genes increase their competitive ability and extend their ecological niches (**Figure 1**). More interestingly, virally encoded host genes also include crucial photosynthetic genetic elements present in cyanophage genomes, which can be used to maintain the targeted function in dead hosts and accomplish the lytic cycle, and can be transferred between hosts as well (Lindell et al., 2005). About 10% of total global photosynthesis could be carried out as a result of phage genes originally from phages (Rohwer and Thurber, 2009). Given the prevalence of phage-encoded biological functions and the occurrence of recombination between phage and host genes, phage populations are thus expected to serve as gene reservoirs that contribute to niche partitioning of microbial species in aquatic ecosystems. Gene transfers by transduction may also represent an important mechanism for gene evolution in natural environments, and bacteriophage transduction could play an important role in contributing to the genetic diversity of microbial populations.

### INDIRECT EFFECTS OF VIRUSES ON MICROBIAL DIVERSITY

The mass release of lytically infected cell contents can change the composition and the bioavailability of ambient organic substrates and nutrients, which are well known as key factors affecting the microbial composition and community structure (**Figure 1**). It has been shown that the presence of grazers in phosphorus-limited microcosms appeared to be a stimulating factor for prokaryotic growth and lytic viral proliferation, with a significant increase in the minor bacterial phylotypes as a consequence of the reduction of resource competition in prokaryotic assemblages (Sime-Ngando and Pradeep Ram, 2005). Prokaryotic phyla belonging to Bacteria, β-Proteobacteria and α-Proteobacteria responded significantly to lysis products, while Archaea and *Cytophaga*-*Flavobacterium* (now known as Bacteroidetes) rather changed their community size structure towards grazing-resistant forms (Pradeep Ram and Sime-Ngando, 2008, 2014). In general, the presence of grazers is a stimulating factor for prokaryotic growth and viral proliferation in the plankton, probably through nutrient regeneration process that increases niche availability and enhances prokaryotic diversity (Pradeep Ram and Sime-Ngando, 2008, 2014). The relative abundance, production and species richness of some bacterial phyla such as *Flectobacillus* or *Actinobacteria* increase more in the presence of both viruses and grazers that when only one of the consumers is present (Simek et al., 2007). Although, Weinbauer et al. (2007) reported both synergistic and antagonistic effects of viral lysis and protistan grazing activities on bacterial biomass, production, and diversity, and considered these effects as the result of group- or species-specific competition for prey and hosts, and the fact that both types of predators produce organic matter that potentially fuel growth.

Lysis products can contain phage-borne enzymes which can kill cells and also influence microbial composition (Fuhrman and Noble, 2000). Lysis products also include free genetic materials that can increase the amount of information encoded in genomes in the water column, and favor horizontal gene transfer mechanisms such as conjugation, transformation or genetic recombination (Weinbauer, 2004). Because all of the direct and indirect roles of viruses in LGTs ultimately result in “novel” genetic materials and information moving into the host cell, there are strong interactions between lateral and vertical gene transfer mechanisms. The acquisition of new genes can affect the genome size and the generation time of host communities and, perhaps more importantly, can move the strain or species barriers in microbial communities (Weinbauer and Rassoulzadegan, 2004). This can also affect the metabolisms and physiology of the host and, hence, their susceptibility to viral infection can be weakened up to a total resistance against viral infections. We are thus in the presence of an effective host-pathogen arms race for survival and coexistence, where host resistance is crucial for offspring and maintenance (**Figure 1**).

## REISTANCE OF VIRAL HOST CELLS

Hosts and pathogens persist in the environment mainly through a molecular arms race between competing hosts and viruses where, as already discussed, viruses can affect their host in various ways, ranging from the enhancement of the host fitness and metabolic performances to mortality. Because parasites and pathogens tend to have shorter generation time than their hosts, they should evolve more rapidly and maintain advantage in the evolutionary race between defense and counter-defense. The paradox here is how do victim species survive and even thrive in the face of a continuous onslaught of more rapidly evolving enemies? One of the explanations is that the physiological, mechanical and behavioral costs of defense are lower compared to the cost of attack (Gilman et al., 2012). The viral host communities can thus respond to the pressure from their parasites and develop a sophisticated resistance shield, specific to each step in the viral cycle, but suffer from the cost of resistance which mainly includes a decrease in the fitness and growth rate or adaptive responses (review in Thomas et al., 2011), such as the production of proteins or extracellular matrices that mask the phage receptor (Bohannan and Lenski, 2000).

Viral receptors on the cell surface are complex families of proteins, carbohydrates or lipids, which serve normal physiological functions of the cells but are hijacked by viruses for their adsorption. Host cell mutations and resistance to viral adsorption grossly include modifications of the receptor structure, alterations of receptor accessibility, decreases in the number of receptors, or loss of receptor sites. A spectacular example of host surface modification is the so-called “Cheshire Cat” strategy where the resistant haploid phase of the algal haptophyte *E. huxleyi* does not calcify and is “invisible” to viruses, in contrast to the susceptible diploid phase (Frada et al., 2008). Similarly, it was shown that colonial forms of the algal prymnesiophyte *P. pouchetii* are resistant to viruses because they are surrounded by an “outer skin,” in contrast to individual cells (Brussaard et al., 2007; Jacobsen et al., 2007). Brussaard et al. (2005) demonstrated that the morphology (solitary versus colonial) of the prymnesiophyte *Phaeocystis globosa* differently regulate viral control of *P. globosa* bloom formation, depending on irradiance, nutrient, and grazing regimes. A modeling exercise suggested that the enhanced growth rates, the low viral infection rate, and the low grazing rate on cells in colonies, as compared to free-living single cells of *P. globosa*, result in a massive blooming of *P. globosa* colonies. When the controlling nutrient becomes depleted, the colonies disintegrate and liberate single colonial cells that are subject to high rates of viral infection and grazing (Ruardji et al., 2005). It is thus likely that cell hosts embedded in colonies are more resistant to viral infection than free-living cell hosts.

The host mechanisms of blocking viral replication and entry remain relatively unknown in marine organisms. Stolt and Zillig (1994) reported a prophage-encoded gene (*rep*) in the marine archaea *Halobacterium salinarum* able to protect cells from viral infection. Tomaru et al. (2009) have shown that viral genome replication in resistant cells of the dinoflagellate *Heterocapsa circularisquama* is repressed. Bacteria, cyanobacteria, and archaea are known to prevent viral replication by the acquisition of immune systems consisting of short fragments of foreign nucleic acids into clustered interspaced short palindromic repeats (CRISPRs) in their genomes. CRISPR spacers have homologies with mobile genetic elements such as bacteriophages and plasmids, and those identical to phage sequences provide resistance against viral infection (Thomas et al., 2011). Overall, the various effects of viruses force costly resistance mechanisms in their host communities, where the co-existence of sensitive and resistant host cells is likely a result of a trade-off between competitive ability and mortality. Lennon et al. (2007) provided a nice example of the ability of marine cyanobacteria *Synechococcus* to evolve resistance with fitness costs associated with the identity of a few particular viruses, suggesting that variability in fitness costs associated with viral resistance can structure microbial communities and regulate biogeochemical cycles.

## VIRUSES AS CELL PARTNERS AND CELL MANIPULATORS

Although viruses are most often studied as pathogens, many are beneficial to their hosts, providing essential functions in some cases and beneficial functions in others. For example, many pathogenic bacteria produce a wide range of virulence factors that help them to infect their hosts. There are numerous examples of such virulence factors that are expressed not from the bacterial genome but from a phage genome, such as diphtheria, Shiga, and cholera toxins (Brüssow et al., 2004). The nuclei of dinoflagellates contain permanently condensed, liquid crystalline chromosomes that seemingly lack histone proteins, and contain remarkably large genomes. The molecular basis for this organization was recently provided by Gornik et al. (2012) who discovered that histone proteins were replaced, during evolutionary events, by a novel, dominant family of nuclear proteins (called DVNPs, dinoflagellate/viral nucleoproteins) that is only found in dinoflagellates and, surprisingly, in a family of large algal viruses, the Phycodnaviridae. The authors concluded that gain of a major novel family of nucleoproteins from an algal virus occurred early in dinoflagellate evolution and coincided with rapid and dramatic reorganization of the dinoflagellate nucleus.

Although few examples for viral manipulators were reported in the microbial world, studies from eukaryotes have shown that some viruses are essential for the survival of their hosts; others give their hosts a fighting edge in the competitive world of nature, while some others have been associated with their hosts for so long that the line between host and virus has become blurred (Roossinck, 2011). Some virologists think that modern genomes are essentially remnants of ancient viruses. Intact and fragmented retroviruses are found in the genomes of almost all eukaryotes. Approximately 8% of the human genome is derived from retroviruses (Lander et al., 2001), and this percentage increases dramatically if other mobile genetic elements are included (Kazazian, 2004). At least some endogenous retroviruses encode functional genes and are thought to be involved in major evolutionary leaps. For example, the evolution of placental mammals probably occurred after the endogenization of a retrovirus. Retroviral envelope proteins cause fusion of cell membranes, a process that not only allows the invasion of oncogenic viruses but also is required for the development of the placental syncytium, an essential part of the barrier that prevents maternal antigens and antibodies getting into the fetal bloodstream and provoking abortion (Dunlap et al., 2006). All living things thus have something of viruses in their genomes, some of which may be beneficial to their fitness and evolution.

Furthermore, recent researches are increasingly demonstrating the ecological and evolutionary importance of viruses of symbiotic organisms within their hosts, in complex tripartite interactions. A famous example is the existence of an endogenous virus that (i) alter the life story of the sea photosynthetic slugs *Elysia chlorotica* by possibly synchronizing the lifetime of the stolen chloroplasts to that of the slugs, (ii) and is probably the vector of the horizontal gene transfer between the slug and the chloroplasts that provides 80–90% of the genes necessary for photosynthesis (Rohwer and Thurber, 2009). However, recent analysis of the genome of *E. chlorotica* egg DNA provides no clear evidence of horizontal gene transfer into the germ line of this kleptoplastic mollus, and suggests that algal nuclear genes or gene fragments are present in the adult slug (Bhattacharya et al., 2013). Polydnaviruses (PDVs) are viruses associated with wasp species that parasitize lepidopteran larvae. PDV particles are injected along with the eggs of the wasp into the lepidopteran larvae (or eggs) and express proteins that interfere with host immune defenses, development, and physiology; this interference enables wasp larvae to survive and develop within the host. It was recently shown that the PDV particles are well conserved in the braconid wasp ovaries and originated from the integration of nudivirus machinery into the genome of an ancestral wasp about 100 million years ago. Bézier et al. (2009) found that nudiviral genes themselves are no longer packaged but are actively transcribed and produce particles used to deliver genes essential for successful parasitism in lepidopteran hosts. Recent works in the field of entomology have revealed tripartite associations between insects, bacteria, and phages, where phages control bacteria which affect the physiology and ecology of the host. Overall, it is clear that viruses are key players in complex symbiotic associations between animals and their sequestered plant chloroplasts, parasitoid insects and their insect hosts, and between bacterial pathogens and their insect hosts. This is an overlooked role of environmental viruses that probably occurs in all habitats where viruses vector important traits, such as defense against or sensibility to parasitoids, within and among symbionts of animal and probably plant host lineages.

## CONCLUDING REMARKS

Aquatic viral ecology is a relatively recent discipline, in increasing development. Viruses are omnipresent in aquatic environments, including the most extreme and worst biotopes, where they often represent the most abundant biological entity. Because all types of cell in the three domains of life have their specific viruses and offer ecological niches to different viral lifestyles, viruses are considered the greatest reservoir of the uncharacterized biological diversity on the earth, which is being probed and described at an increasingly rapid rate, almost exclusively with molecular sequence data. For example, Hewson et al. (2013) recently used a metagenomic approach to identify circular, single-stranded DNA viruses that may be involved in the seasonal dynamics of *Daphnia* spp. in Oneida and Cayuga lakes (upstate New York). Because *Daphnia* plays a critical role in many lake ecosystems, such viruses may have important effects on herbivory and thus carbon flow through the lake ecosystem.

Our conceptual understanding of the function and regulation of aquatic ecosystems, from microbial to global biogeochemical processes, has changed with the study of viruses. Viral-mediated prokaryotic mortality roughly equals bacterivory from protists, which is a significant departure from the traditional view that predation and resource availability are the main factors controlling prokaryotic abundance and production in pelagic systems. Viruses influence both the retention and the export of organic matter in the pelagic realms. Given the prevalence of phage-encoded biological functions within host cells and the occurrence of recombination between phage and host genes, phage populations serve as gene reservoirs that contribute to niche partitioning of microbial species in aquatic ecosystems. Viral-mediated gene transfers include diverse mechanisms (transduction, transformation, conjugation, and recombination) that are known to affect gene evolution in the marine environment. It is thus clear that most of the viruses are not pathogens but mutualistic cell partners that provide helper functions. The discovery of giant viruses of eukaryotes (absent in this review) which encode trademark cellular functions has weakened the gap between inert and living things. Overall, studies in aquatic viral ecology are sources of novel knowledge related to the biodiversity of living things, the functioning of ecosystems, and the evolution of the cellular world. The future challenge is to extend viral ecology studies (i) to all biotopes in the biosphere such as lakes, rivers, underground waters, soils, clouds, air, etc., (ii) to all living organisms which are susceptible to viral attacks (e.g., zooplankton, Archaea etc.), and (iii) the related functions, some of which are crucial to global change (e.g., consumption and production of greenhouse gas such as methane etc.), and (iv) to multi-partner symbioses and their effects on the food web dynamics and host maintenance and adaptive evolution.

More generally, next-generation sequencing technologies are increasingly revealing that microbial taxa likely to be parasites or symbionts are probably much more prevalent and diverse than previously thought. Every well-studied free-living species has parasites; parasites themselves can be parasitized. As a rule of thumb, there is an estimated four parasitic species for any given host, and the better a host is studied the more parasites are known to infect it. Therefore, parasites and other symbionts should represent a very large number of species and may far outnumber those with “free-living” lifestyles. Paradoxically, free-living hosts, which form the bulk of our knowledge of biology, may be a minority. Microbial parasites offer good experimental models because they are typically characterized by their small size, short generation time, and high rates of reproduction, with simple life cycle occurring generally within a single host. They are diverse and ubiquitous in aquatic ecosystems, comprising viruses, prokaryotes, and eukaryotes. Extensive studies on all aspects of parasites and other symbionts in aquatic microbial ecology are warranted, including method development, life cycle, interactions with hosts and competing microbes, coevolution, effects on food webs, and biogeochemical cycles. I believe that including viruses in the more complex world of parasites and symbionts is promising for biology and ecology in the future.

## Conflict of Interest Statement

The author declares that the research was conducted in the absence of any commercial or financial relationships that could be construed as a potential conflict of interest.

## Footnotes

1 http://www.isvm.org/

2 http://www.ictvonline.org/index.asp?bhcp=1
